# QR code model: a new possibility for GPCR phosphorylation recognition

**DOI:** 10.1186/s12964-022-00832-4

**Published:** 2022-03-02

**Authors:** Hao Chen, Suli Zhang, Xi Zhang, Huirong Liu

**Affiliations:** 1 Department of Physiology and Pathophysiology, School of Basic Medical Sciences, Capital Medical University, 10 Xitoutiao, You An Men Street, Beijing, 100069 People’s Republic of China; 2 Beijing Key Laboratory of Metabolic Disorders Related Cardiovascular Disease, Capital Medical University, Beijing, 100069 People’s Republic of China

**Keywords:** GPCR phosphorylation recognition, Bar code model, Flute model, QR code model, GPCR signaling

## Abstract

G protein-coupled receptors (GPCRs) are the largest family of membrane proteins in the human body and are responsible for accurately transmitting extracellular information to cells. Arrestin is an important member of the GPCR signaling pathway. The main function of arrestin is to assist receptor desensitization, endocytosis and signal transduction. In these processes, the recognition and binding of arrestin to phosphorylated GPCRs is fundamental. However, the mechanism by which arrestin recognizes phosphorylated GPCRs is not fully understood. The GPCR phosphorylation recognition “bar code model” and “flute” model describe the basic process of receptor phosphorylation recognition in terms of receptor phosphorylation sites, arrestin structural changes and downstream signaling. These two models suggest that GPCR phosphorylation recognition is a process involving multiple factors. This process can be described by a “QR code” model in which ligands, GPCRs, G protein-coupled receptor kinase, arrestin, and phosphorylation sites work together to determine the biological functions of phosphorylated receptors.

**Graphical Abstract:**

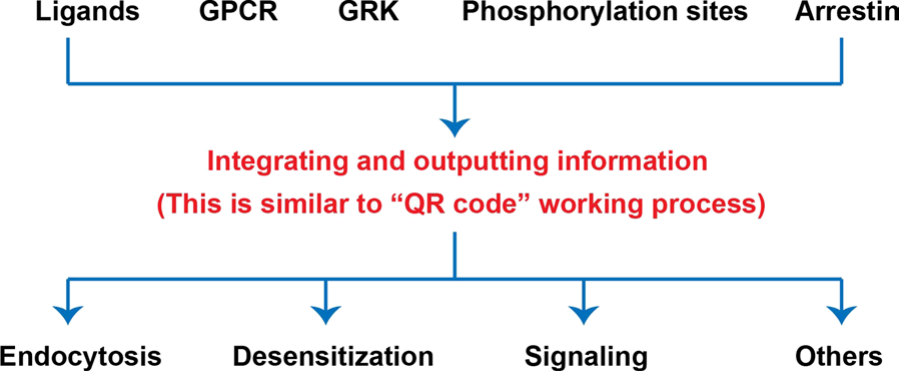

**Video Abstract**

**Supplementary Information:**

The online version contains supplementary material available at 10.1186/s12964-022-00832-4.

## Background

G protein-coupled receptors (GPCRs) are the largest class of 7-transmembrane receptors on human cell membranes and can accurately transmit extracellular information, such as from hormones, neurotransmitters and odors, into the cell. GPCRs are an important information transmission hub [1–3]. There are two main mechanisms that mediate GPCR signaling: the G protein-dependent pathway and the G protein independent pathway [2, 4–10]. The G protein-dependent pathway is mainly regulated by guanine nucleotide-binding proteins (G proteins). G proteins are a class of highly conserved proteins, including Gs, Gi, Gq, and G12, whose main function is to transmit GPCR signals [11–14]. For example, in cardiomyocytes, catecholamine activates β_1_-adrenoceptor (β_1_-AR) and activates the Gs-adenylate cyclase (AC)-cyclic adenosine monophosphate (cAMP)-protein kinase A (PKA) pathway to exert positive chronotropic and inotropic effects. In heart failure, the release of a large amount of catecholamine and activation of the β_2_-adrenoceptor (β_2_-AR)-Gi pathway can inhibit the activity of AC and weaken β_1_-AR-Gs activation [15–18]. The G protein-independent pathway is mainly regulated by arrestin that recognizes and binds phosphorylated GPCRs catalyzed by G protein-coupled receptor kinase (GRK). Arrestin binding to a phosphorylated GPCR prevents G proteins from binding the GPCR and promotes receptor internalization [11, 19–22]. Arrestin binding to GPCRs can also transmit cellular signals. The ability of arrestin to recognize and bind phosphorylated GPCRs is the basis for achieving GPCR signaling. However, the specific mechanism by which this occurs is not completely clear.

Previous studies have found that rhodopsin phosphorylation can enhance arrestin binding [23]. It was further found that arrestin mainly recognizes the phosphorylated C-terminus of GPCRs [24]. The GPCR C-terminus phosphorylated by different protein kinases can transmit different downstream signals. To describe this, a “bar code” model [25, 26] has been proposed. The bar code model has given new insights into GPCR phosphorylation recognition, and the theory of the model has been continuously improved in several follow-up studies. Based on the bar code model, the “flute” model integrates structural changes of β-arrestin with GPCR phosphorylation recognition patterns. Different GPCR phosphorylation sites match different arrestin structures, and this matching determines the resulting signaling [27, 28]. Recently, molecular dynamics simulations and amino acid site mutations were used to verify the function of the vasopressin type 2 receptor (V_2_R) phosphorylation site in binding and activating β-arrestin. The different combinations of GPCR phosphorylation sites were found to directly affect the structural changes of β-arrestin and its binding to the receptor. This finding also indicated the diversity of the structural changes of β-arrestin [29, 30]. In a study of V_2_R, Qing-Tao He et al. analyzed the structure of the complex comprising the V_2_R C-terminus and β-arrestin1. The authors found that phosphorylation at different sites of V_2_R causes different structural changes in arrestin, which alters β-arrestin1 function [31]. A series of recent studies on bar code models and flute models have analyzed the phosphorylation recognition patterns of GPCRs and arrestin from the perspective of protein structure, bringing us closer to a comprehensive understanding of phosphorylation recognition patterns.

The purpose of this review was to discuss the mechanism by which arrestin recognizes GPCR phosphorylation, analyze the effects of the factors involved in this process (ligands, GPCR type, GRK, arrestin and GPCR phosphorylation sites), and briefly describe the historical development of phosphorylation recognition research.

## Factors in GPCR phosphorylation recognition

The process of GPCR phosphorylation recognition is extremely complex and delicate. Ligands, GPCR types, GRK, arrestin, GPCR phosphorylation sites can all influence or even determine which function the receptor performs. Therefore, we first illustrate the effects of different factors on GPCR phosphorylation recognition.

### Ligands

Ligands are the drivers of GPCR signaling. Light, odors, peptides, ions, hormones and antibodies are all become GPCR ligands [32, 33]. For example, light can activate rhodopsin in rod cells [34]. Angiotensin II type I receptor (AT1R) autoantibodies (AT1-AA) can continuously activate AT1R in vascular smooth muscle cells [35], and catecholamines can activate adrenaline receptors in cardiomyocytes [36]. GPCRs are also the target of many drugs. Some drugs, such as atropine, scopolamine (acetylcholine muscarinic receptor blocker), propranolol (β-adrenoceptor blocker), losartan (AT1R blocker) and phentolamine (alpha-adrenoceptor blocker), act by binding to GPCRs [37]. These are examples of drugs that are commonly used in the clinic [38–41].

**Fig. 1:**
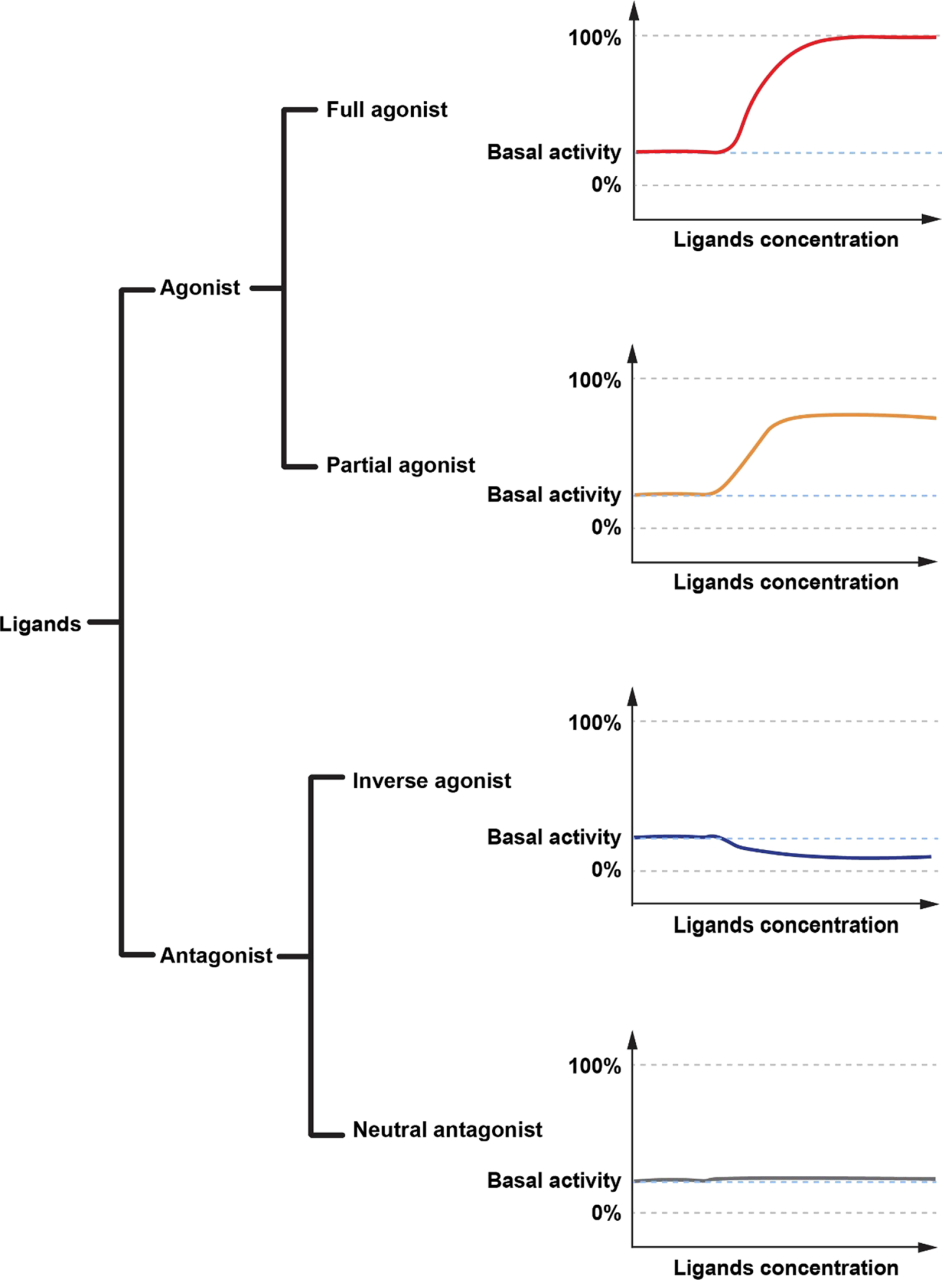
Classification of GPCR ligands based on the effect of GPCRs active state. Complete agonists can maximize GPCR activation and signal transmission. Partial agonists can activate receptors but cannot maximize receptor activation. Inverse agonists inhibit the basal activation of the receptor. Neutral antagonists inhibit receptor activation but do not affect basal activation

Ligand classification is a complex matter. Based on the effect of GPCRs active state, ligands can be divided into agonists and antagonists (Fig. 1) [37]. According to their degree of GPCR activation, agonists can be divided into complete agonists and partial agonists. Complete agonists maximize the activation of GPCRs and result in the strongest signal transduction. PNU282987, a complete agonist of α7 nicotinic receptor (α7 nAChR), reverses depressive symptoms in Sprague Dawley (SD) rats induced by chronic mild stress [42]. Formoteru, a complete agonist of β_2_-AR, is used to treat asthma because of its bronchodilation effect [43]. Partial agonists partly activated a certain pathway when compared with the efficacy of a complete agonist. Δ9-Tetrahydrocannabinol (Δ9-THC) is a partial agonist of cannabinoid receptor 1 (CB1). It plays a role in the treatment of mental illnesses by activating CB1 [44, 45]. Antagonists are divided into inverse agonists and neutral antagonists. Inverse agonists inhibit basal activation of the receptor, reducing activation to lower than the basal level. Basal activation is inherent in some GPCRs and does not require ligand involvement. Neutral antagonists inhibit the effect of agonists but do not affect basal activation [37, 46].

However, the same ligand that plays a role of agonist in a response may become antagonist or inverse agonist in another response. This complicacy is the pluridimensional efficacy of a ligand [47]. For inverse agonist, the efficacy defined the ability to change receptor behavior become vectorial [48]. For example, carvedilol, as inverse agonist for β-adrenergic receptor, inhibited the production of cAMP medicated by Gs in congestive heart failure. However, carvedilol played the role of partial agonist for the activation of extracellular regulated protein kinases (ERK)1/2 mediated by β-arrestin [49, 50]. Similarly, [D-Trp12, Tyr34]-bPTH, the ligand of parathyroid hormone (PTH) receptor, was classified as antagonist of calcium signaling and an inverse agonist for cAMP production. However, it was also classified as partial agonist for ERK1/2 signaling [51, 52].

Topographically, according the variant of binding sites, the GPCR ligands are divided into orthosteric ligands and allosteric ligands (also named allosteric modulators) [53]. The ligands that bind to the endogenous ligands (hormone or neurotransmitter) binding sites (orthosteric sites) are orthosteric ligands [54]. Allosteric ligands bind to recognition sites that are distinct from the orthosteric sites [55]. The allosteric ligands are divided into four categories, including negative allosteric modulators (NAMs), positive allosteric modulators (PAMs), allosteric agonists and silent allosteric modulators (SAMs). Ligands that bind to an allosteric site of the receptor resulting in inhibition of receptor function are considered NAMs. Because NAMs decrease the affinity of orthosteric agonist ligands by changing receptor conformation. PAMs have the opposite effect to NAMs. In the absence of orthosteric agonist, allosteric agonist can active GPCR by itself. SAMs bind to allosteric sites on the receptor but do no affect receptor function [56]. In the development of GPCR drugs, allosteric ligands have drawn more and more attention, especially for small molecules [57, 58]. For example, maraviroc was the allosteric modulator for chemokine receptor CCR5. The function of maraviroc was to treat Acquired Immune Deficiency Syndrome (AIDS) by allosteric inhibition of CCR5 chemokine signaling and the prevention of human immunodeficiency virus type 1 (HIV-1) entry [59].

The ligand is the initiating factor in GPCR signaling. The type and characteristics of a ligand will specifically change the structure of a GPCR, impacting the final biological results [60]. The complex and diverse array of ligands are the initial factors affecting the process of receptor phosphorylation.

### GPCR type

Based on the homology of the GPCR sequence, receptors can be divided into class A (rhodopsin family), B (secretin family), C (glutamate family), the adhesin family, and class F (frizzled family) [61]. This large number of GPCRs constitutes a broad signal regulation network throughout the body that transforms external information into biological information and transmits it through signaling pathways [62].

GPCRs are widely distributed throughout the body, and their signal regulation, including phosphorylation, is extremely complex. The phosphorylation of GPCRs is the key to ensuring accurate receptor function. In the heart, catecholamines activate β_1_-AR to exert positive chronotropic and positive inotropic effects through the cAMP/PKA pathway. To avoid the overactivation of β_1_-AR, β_1_-AR is phosphorylated by GRK in a timely manner. β-Arrestin binds to phosphorylated β_1_-AR to assist receptor desensitization and endocytosis [16]. If GPCRs are not phosphorylated or if they are hyperphosphorylated, their signal can be disturbed, leading to the occurrence and development of disease. For example, GRK2 was a phosphatase in M2 macrophages. The downregulation of GRK2 expression overactivated the β_2_-AR/cAMP/PKA pathway in hepatocellular carcinoma, thereby promoting the growth of cancer cells [63]. GRK4-induced hyperphosphorylation of adiponectin receptors can lead to the development of hypertension [64]. These studies suggest that timely and appropriate phosphorylation ensures the accurate transmission of extracellular information by GPCRs and maintains homeostasis.

### GRK

As an integral part of GPCR phosphorylation, the function of GRK is to phosphorylate the receptor. GRK catalyzes receptor phosphorylation, which enables the receptor to bind to arrestin and carry out its biological function. GRK is serine/threonine protease [65]. GRK was initially elucidated as an enzyme that promotes rhodopsin phosphorylation in a light-dependent manner. This enzyme was termed rhodopsin kinase (GRK1) [66]. Subsequently, β_2_-adrenoceptor kinase (GRK2) was found in a study of β_2_-AR [67]. To date, researchers have discovered seven GRKs (GRK1-GRK7) [66–73]. Among them, GRK2, 3, 5, and 6 are widely distributed throughout the body and regulate the phosphorylation process of most GPCRs [65, 74]. GRK1 and 7 are mainly distributed in the visual system and function in visual regulation. GRK1 is distributed in cones and rods, while GRK7 is mainly distributed in cones [75]. GRK4 is found in the testis [74].

The differences in GRK distribution means that different GRKs can only interact with tissue distribution-specific GPCRs. GRK1 mainly phosphorylates rhodopsin in rod cells [76]. GRK7 mainly compensates for the deficiency of GRK1 function in Oguchi patients [75]. Although GRK2, 3, 5, and 6 can interact with the vast majority of GPCRs, the effect of GPCR phosphorylation is not consistent. For example, GRK2 and GRK3 promote the desensitization of phosphorylated V_2_R, while GRK5 and GRK6 catalyze V_2_R phosphorylation and continue to transmit information to the ERK signaling [77]. Similarly, GRK2 and GRK3 catalyze AT1R phosphorylation to promote receptor desensitization and endocytosis, while GRK5 phosphorylates AT1R, leading to β-arrestin-dependent ERK activation [78]. The reason for this phenomenon may be related to the phosphorylation of different sites in GPCRs being catalyzed by different GRKs [27]. Thirteen serine (Ser)/threonine (Thr) residues (Ser246, Ser261, Ser262, Ser345, Ser346, Ser355, Ser356, Thr360, Ser364, Ser396, Ser401, Ser407, and Ser411) of β_2_-AR were identified by small interfering RNA (siRNA) and quantitative mass spectrometry. GRK2 mainly phosphorylated Thr360, Ser364, Ser396, Ser401, Ser407, and Ser411, while GRK6 phosphorylated only Ser355 and Ser356. This difference in phosphorylation sites eventually leads to the β_2_-AR-β-arrestin-ERK1/2 pathway being catalyzed by GRK6, while the phosphorylation of β_2_-AR catalyzed by GRK2 inhibits this signaling pathway [79]. These results suggest that different GRKs have different preferred phosphorylation sites and relatively fixed functions. GRK2-catalyzed receptor phosphorylation tends to promote endocytosis, while GRK6-catalyzed receptor phosphorylation tends to induce β-arrestin-dependent signal transduction.

**Fig. 2:**
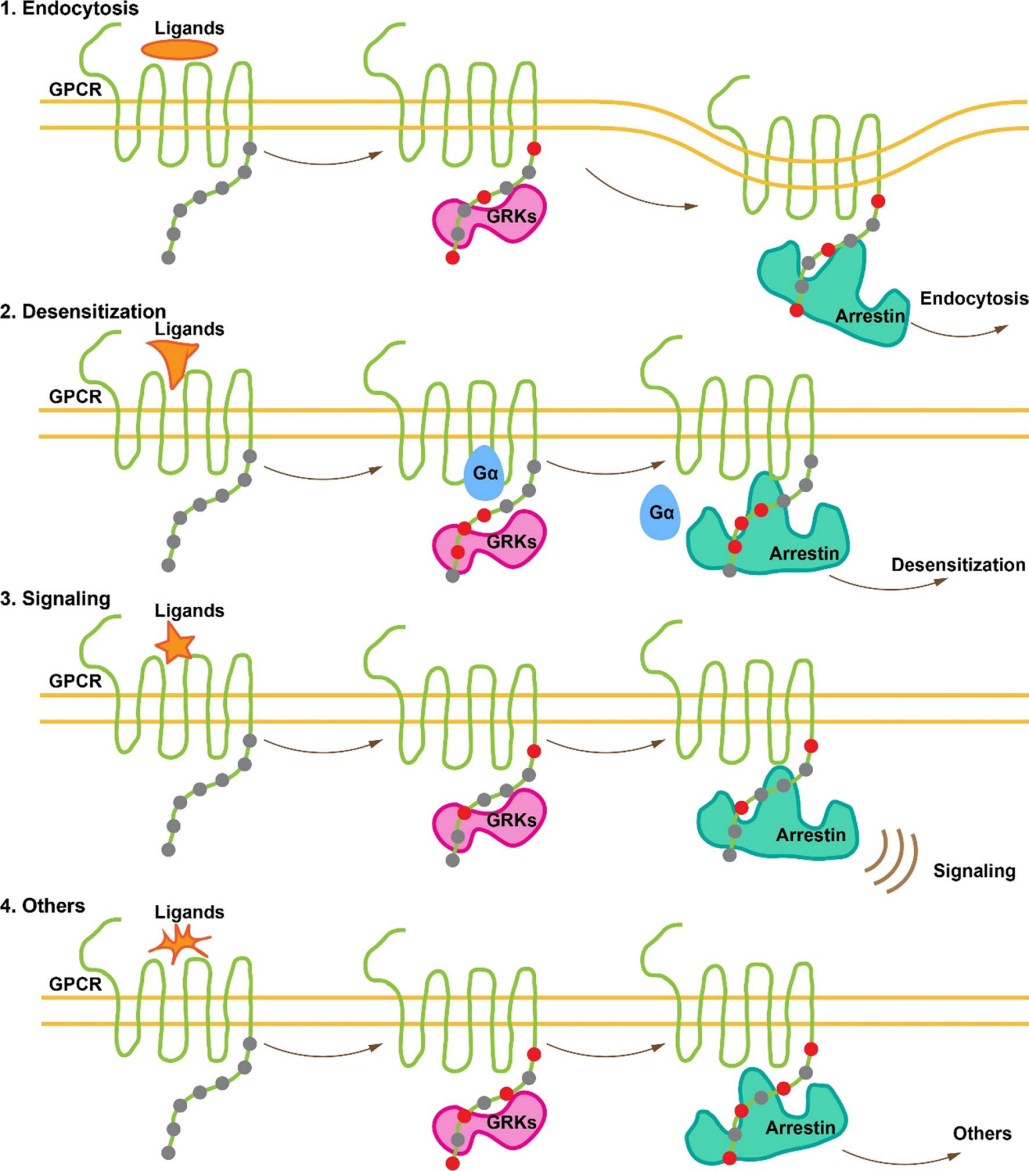
Ligand-GPCR-GRK-arrestin information transfer process. After the ligand activates the GPCR, GRK phosphorylates the activated receptor. Arrestin recognizes the phosphorylated receptor and binds to it, thereby causing receptor desensitization, endocytosis or the continuation of signal transmission. In this process, different GRKs phosphorylate different sites on the receptor. Arrestin recognizes these differences in phosphorylation sites and binds to the receptor in different structural conformations to perform different functions. The orange shapes represent different ligands, the gray dots represent sites that are not phosphorylated, and the red dots represent sites that are phosphorylated.

The rich combination of seven GRKs, different tissue distributions and different preferences for phosphorylation sites allows a complex array of signaling that is regulated by GPCR phosphorylation. This complexity can accurately meet the signaling requirements of the body (Fig. 2). The diversity of phosphorylation site combinations also leads to different binding patterns of arrestin.

### Arrestin

There are four types of arrestin: arrestin1, arrestin2, arrestin3, and arrestin4. Similar to GRK, the distribution of arrestin is tissue-specific. Arrestin1 and 4, also known as visual arrestins, are mainly distributed in the visual system; arrestin2 and 3 (β-arrestin1 and 2) are also widely distributed in the visual system [80]. Four types of arrestin have similar structure. Briefly, two crescent-shaped beta-sandwiches (N-and C-terminal domains, respectively) made up the structure of arrestin. The central crest, including finger loop, middle loop and C-loop, formed between the above domains. Figure loop was a key receptor-binding element. In the inactive state, the structure of finger loop was irregular. However, by interacting with the GPCR, α helix was formed in finger loop to assist arrestin to interact with phosphorylated GPCR easily [81]. The function of middle loop and C-loop was to stabilize the inactive basal-state arrestin. Besides, the basal structure of arrestin was also stabilized by the gate loop which consisted of C-terminal and polar core [81, 82].

Arrestins are multifunctional regulators of GPCR. When arrestin was discovered, its only function was identified as assisting in the desensitization of activated GPCRs [83, 84]. Further research has gradually discovered additional functions of arrestin. At present, it is believed that arrestin has three main functions: (1) assisting in the desensitization of activated receptors; (2) mediating receptor endocytosis; (3) signaling [85]. The basis for the above regulatory functions of arrestin lies in the changes in the structure of arrestin and the conformation with which it binds to the GPCR [86]. In the process of receptor desensitization, arrestin binds to GPCR transmembrane core, putting itself in a conformation named “core” conformation. G protein is excluded from the receptor by arrestin, thereby terminating the signal transduction of the G protein pathway [20]. In the process of receptor endocytosis, arrestin binds to phosphorylated GPCR C-terminal with “tail” conformation [21]. Arrestin recruits intracellular clathrin and β2-adaptin (AP2) in the form of cytoskeletal proteins to form endosomes and promote receptor internalization. Endocytosis receptors enter the lysosome and are either degraded, or they returned to the cell membrane to again carry out their function [87]. Arrestin can be considered as a transit point for GPCR signaling. During the signaling process, arrestin remains bound to the GPCR by “tail” conformation. Typically, activation of the SRC is seen in GPCR signaling via arrestin. Compared with the administration of β-arrestin1 alone, β-arrestin1 binding to phosphorylated β_2_-AR significantly increased the phosphorylation of SRC [9]. Biased activation of AT1R-β-arrestin1 promotes acute catecholamine secretion by recruiting transient receptor potential cation channel subfamily C 3 [88]. Besides, the mitogen-activated protein kinase (MAPK), tyrosine kinase Ser-Thr kinase Akt pathways are also transmitted by GPCR bound to arrestin and are used to regulate cell proliferation, migration, and apoptosis [89].

Regardless of which function arrestin performs, the specific combination of phosphorylated GPCRs is a prerequisite. The four classes of arrestin and their distribution characteristics form the basis of its finely tuned regulation of the GPCR signaling pathway. The four types of arrestin have similar structures and amino acid sequences [87, 90], but their differences in distribution and function suggest that there may be differences in the process of phosphorylation recognition. In the visual system, the homology between arrestin1 and arrestin4 is approximately 58%. Arrestin1 is mainly distributed in rod cells and binds to light-activated phosphorylated rhodopsin, while arrestin4 is only found in cones and has the highest affinity for human green cone opsin [91, 92]. Arrestin1 can inactivate both rhodopsin and cone pigment, while arrestin4 can inactivate only cone pigment [93–95]. Although arrestin2 and arrestin3 have high homology (76% identical) [92], they have different affinities for different combinations of GPCR phosphorylation.

According to their differences in affinity for arrestins, GPCRs can be divided into two types: type A and type B. Type A GPCRs do not interact with visual arrestin, and their affinity for β-arrestin2 is higher than that for β-arrestin1. Examples of type A GPCRs include β_2_-AR, μ opioid receptors, endothelin receptor A, dopamine D1A receptor, and α_1_ adrenoceptor. Type B GPCRs interact with visual arrestin, and there is no difference in affinity for β-arrestin1/2; examples of type B GPCRs include AT1R, neurotensin receptor 1, V_2_R, and thyrotropin releasing hormone receptor [96]. Although arrestins have similar structure and function in a broad sense, the difference between them may be the crux to affect the different functions for each type of arrestin. However, at present, the understanding of GPCR-arrestin phosphorylation coding is mainly based on arrestin2 (β-arrestin1) [27, 29, 97]. Therefore, our understanding of the phosphorylation recognition pattern needs to be further clarified.

Recently, Latorraca et al*.* and Dwivedi-Agnihotri et al*.* found that when binding different phosphorylation sites at the C-terminus of GPCRs, arrestin undergoes different structural changes, and the structural changes of one region are not necessarily accompanied by structural changes in other regions [29, 30]. For example, the phosphorylation of Ser350 in V_2_R caused structural changes in the β-arrestin1 gate loop and finger loop but had little effect on the C-terminal structure of β-arrestin. However, during the phosphorylation of Ser360 in V_2_R, the change in the C-terminal structure of β-arrestin was significantly greater than that of the gate loop and finger loop [29]. This discovery contrasted with the original view that the structural changes of arrestin switch between an activated state and an inactive state [26, 29]. The diversity of structural changes may be one of the reasons why only four types of arrestin are needed to regulate the function of more than 800 GPCRs.

In summary, although there are only four types of arrestin, they can sufficiently and accurately transmit complex information to cells. This is mainly due to the diversity in the distribution, function, and structural changes of arrestins (Fig. 2). This diversity is fundamental to the process of arrestin’s recognition of phosphorylation.

### GPCR phosphorylation sites

The phosphorylation sites of GPCRs are mainly located in the third intracellular loop and at the C-terminus. The arrestin binding sites are primarily found in the C-terminus of GPCRs. At these sites, serine (Ser, S) and threonine (Thr, T) form the S/T amino acid cluster. The function of the S/T cluster is to stabilize the binding between GPCRs and arrestin [98, 99] and to promote GPCRs to complete arrestin-clathrin-dependent endocytosis [100]. However, the number of phosphorylation sites differs among GPCRs. For example, neurotensin receptor subtype 1 (NTS1) has six phosphorylation sites in the C-terminus; AT1R, CXCR2 and CXCR5 all have seven phosphorylation sites; and CXCR3 and CCR7 have eight phosphorylation sites [98].

In the process of GPCR–arrestin interaction, different phosphorylation sites have different functions (Fig. 2). There are eight phosphorylation sites at the C-terminus of V_2_R. Using molecular dynamics simulation, researchers found that the Ser350 phosphorylation site promotes the activation of β-arrestin1, while the Thr360 site contributes to the binding of β-arrestin1 to V_2_R. However, when Ser350 and Ser362 were jointly phosphorylated, the binding ability of β-arrestin1 decreased significantly. Similarly, when Ser357 and T360 were jointly phosphorylated, the activation ability of β-arrestin1 also decreased significantly [29]. This suggests that the quality of a phosphorylation site might be more important to the interaction between a GPCR and arrestin than the number of phosphorylation sites. This conclusion was supported by experiments that investigated the phosphorylation sites of V_2_R by amino acid mutation [30]. Thr347 and Ser350 were not necessary for the recruitment of β-arrestin. Ser357 affected the recruitment and migration of β-arrestin but had no effect on the activation of ERK. Both Ser362 and Ser363 affected the recruitment of β-arrestin, and the common influence of these two sites was significantly stronger than that of each single site alone. When S362 and S363 were mutated to alanine (Ala, A), V_2_R lost its ability to recruit β-arrestin and become a G protein-biased receptor. Thr360 played a key role in recruiting V_2_R and β-arrestin, determining β-arrestin migration, and activating ERK, but the role of Thr359 in these functions was much smaller.

The phosphorylation of different sites is mainly affected by GRK. GRK2 and GRK6 can cause V_2_R to form different phosphorylation combination patterns. Β-Arrestin1 recognizes difference in phosphorylation patterns and determines downstream signaling [27].

## Exploration of GPCR phosphorylation recognition by arrestin

### Early exploration

The C-terminal phosphorylation of GPCRs is the basis of arrestin binding [101, 102]. However, this was not clear in the early stages of elucidating the function of arrestin. In 1984, researchers found that the binding of arrestin to rhodopsin is strictly based on the light-dependent mode, that is, rhodopsin is phosphorylated and binds arrestin as a result of light activation. However, in the absence of light activation, the two proteins do not bind. This suggested that the phosphorylation of rhodopsin enhances arrestin binding. This was also the initial understanding of GPCR phosphorylation and arrestin binding [23]. Using limited proteolysis and other methods, Krzysztof Palczewski and colleagues found that the 163–182 amino acid residues of arrestin constitute a region that recognizes phosphorylated rhodopsin [103]. This was basically consistent with the findings of Vsevolod V. Gurevich and Jeffrey L. Benovicarrestin who used arrestin complementary deoxyribo nucleic acid (cDNA) truncation to modify arrestin expression and found that the region in arrestin that recognizes the C-terminus of phosphorylated rhodopsin is composed of amino acid residues 158–185 [24].

In 1987, with the discovery of visual arrestin analogs, the understanding of arrestin gradually expanded from rhodopsin to all GPCRs and made it clear that arrestin binding requires GPCR phosphorylation [104]. Using amino acid mutation techniques, Sergey A. Vishnivetskiy and others found that the polar core in arrestin plays an important role in the recognition of phosphorylated rhodopsin. The polar core is composed of Arg175, Asp30, Asp296, and Asp303, in which the function of Arg175 is a phosphorylation-sensitive trigger. The role of the polar core is to stabilize arrestin in the inactive state. The phosphorylated rhodopsin can bind to Arg175 and break the electrostatic stability of the polar core, which promotes the transformation of arrestin from an inactive state to an activated state [105].

These studies suggest that GPCR phosphorylation is a prerequisite for arrestin binding and the transduction of signal through the G protein-independent pathway. In this context, the role of GPCR phosphorylation recognition patterns in signal transduction must be clarified. With a further in-depth understanding of GPCR phosphorylation and arrestin binding, researchers have proposed and refined the GPCR phosphorylation recognition bar code model and flute model.

### GPCR phosphorylated bar code model

**Fig. 3:**
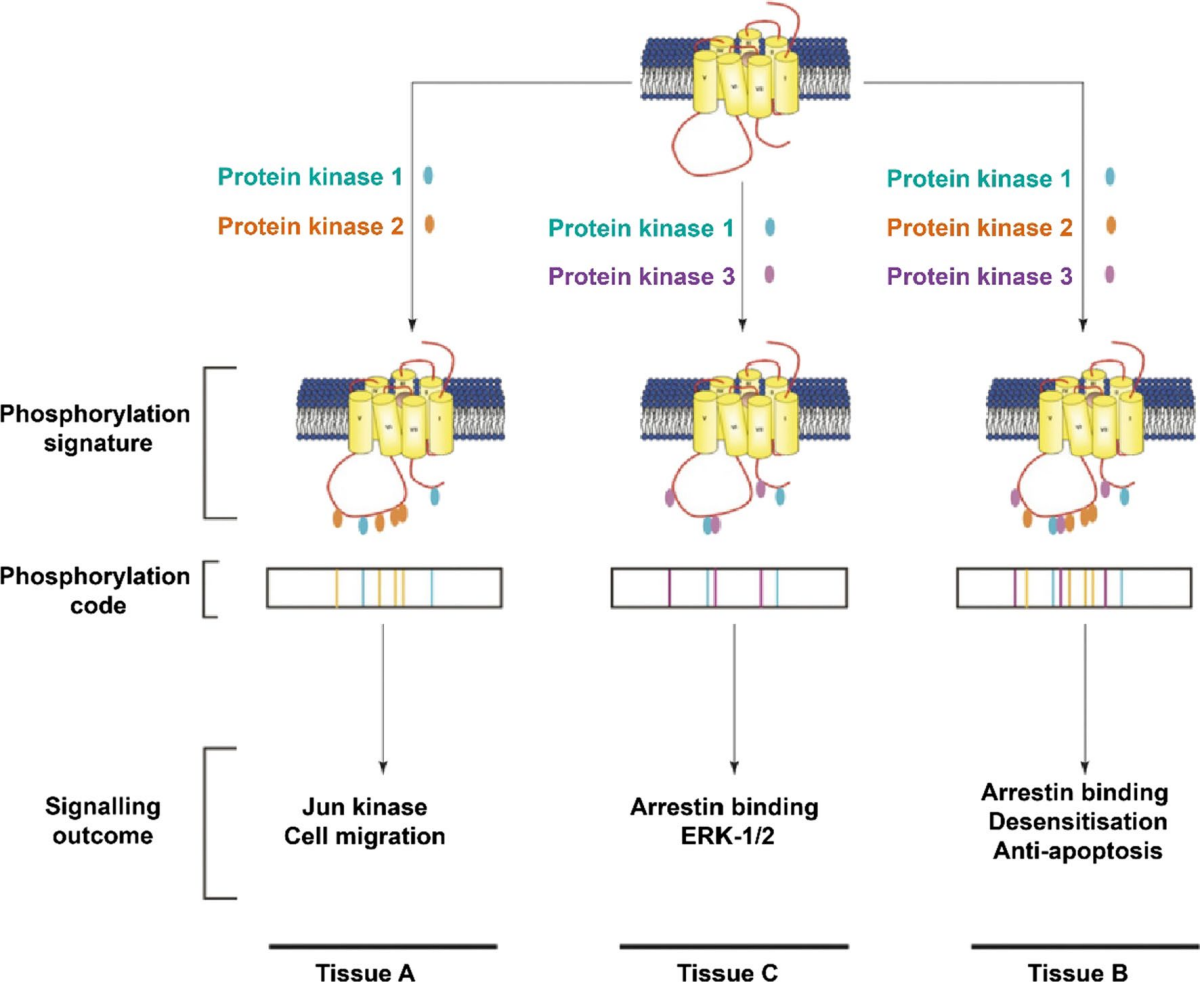
Bar code model diagram. This picture is from the review by Tobin et al. [25]

Previous studies have found that GPCRs phosphorylated by different protein kinases can have different biological functions. For example, β_2_-AR phosphorylation catalyzed by GRK promotes β-arrestin binding to the receptor, while phosphorylation by PKA does not result in β-arrestin recruitment. However, both kinases cause β_2_-AR desensitization [106–110]. Protein kinase CK2 activates the ERK and Jun kinase pathways after the phosphorylation of M3 muscarinic receptors [111]. To explain these phenomena, Andrew B. Tobin et al. proposed a phosphorylated bar code model (Fig. 3) to elaborate the relationship between GPCR phosphorylation and downstream signaling. The bar code model proposes that different protein kinases catalyze the phosphorylation of GPCRs at different sites, resulting in the activation of different signaling pathways [25, 26]. When the bar code model was initially proposed, receptor phosphorylation was not limited to the activity of GRK, and the signal transduction process did not only occur through arrestin. In later research, the phosphorylation recognition model tended to state that GRK catalyzed receptor phosphorylation, and signal transduction was mediated by arrestin.

In 2017, X. Edward Zhou et al*.* reported an activated rhodopsin-arrestin complex. Two phosphorylation coding modes for the combination of GPCR and arrestin, namely, “PXPXXP/E/D” and “PXXPXXP/E/D”, were identified. P represents phosphorylated serine or threonine, X represents any amino acid residue, except that proline cannot be present at either of the second XX sites. When the GPCR C-terminus exhibits this pattern of phosphorylated amino acids, it is recognized by arrestin, which then binds to it. In an analysis of 825 GPCRs, one or two phosphorylation coding modes were found in 436 GPCR C-termini. These two phosphorylation coding patterns may therefore be universal arrestin recognition codes of phosphorylated GPCRs [97]. This finding defined the environment of GPCR phosphorylation sites recognized by arrestin.

Recently, researchers have used molecular dynamics simulations and site-directed spectroscopy to analyze phosphorylated V_2_R binding to activated β-arrestin1 [29]. The results revealed several aspects of GPCR–arrestin binding. (1) The binding of arrestin to GPCRs depended on the spatial arrangement of phosphorylated amino acids rather than on the number of phosphorylated amino acids. Simulating the phosphorylation of all 8 phosphorylation sites at the V_2_R C-terminus (Thr347, Ser350, Ser357, Thr359, Thr360, Ser362, Ser363, Ser364) promoted the binding and activation of β-arrestin1. The same effect could be achieved by phosphorylating the second site (pSer350, p2) alone. However, phosphorylation of the third site (pSer357, p3) alone or the simultaneous phosphorylation of the third and fifth sites (pSer357 + pThr360, p3, 5) did not induce β-arrestin binding and activation. (2) The phosphorylation patterns of GPCRs that favor arrestin binding were different from the phosphorylation patterns of GPCRs that favor arrestin activation. Among the eight phosphorylation sites of V_2_R, pThr360 (p5) simultaneously favored β-arrestin1 binding and activation. pSer350 (p2) and pThr359 (p4) mainly favored β-arrestin1 activation. pSer363 (p7) mainly favored β-arrestin1 binding. (3) Arrestin has a complicated spatial structure, and the structural changes of each part were not synchronized upon GPCR binding and activation. When the gate loop of β-arrestin1 was in an inactive state, the finger loop was able to be in an activated state that bound to V_2_R. (4) Different patterns of phosphorylation led to a variety of arrestin structures. The study found that arrestin has multiple forms after binding to GPCRs, and the form depends on the spatial arrangement of the phosphorylation sites and charge attraction. These findings enrich our understanding of the active and inactive states of arrestin. This research extended the study of the bar code model from two-dimensional space to three-dimensional space and clarified the structural basis of the bar code model. However, the way in which complex phosphorylation coding and arrestin spatial structures correspond to downstream signaling requires further study.

### GPCR phosphorylation flute model

**Fig. 4:**
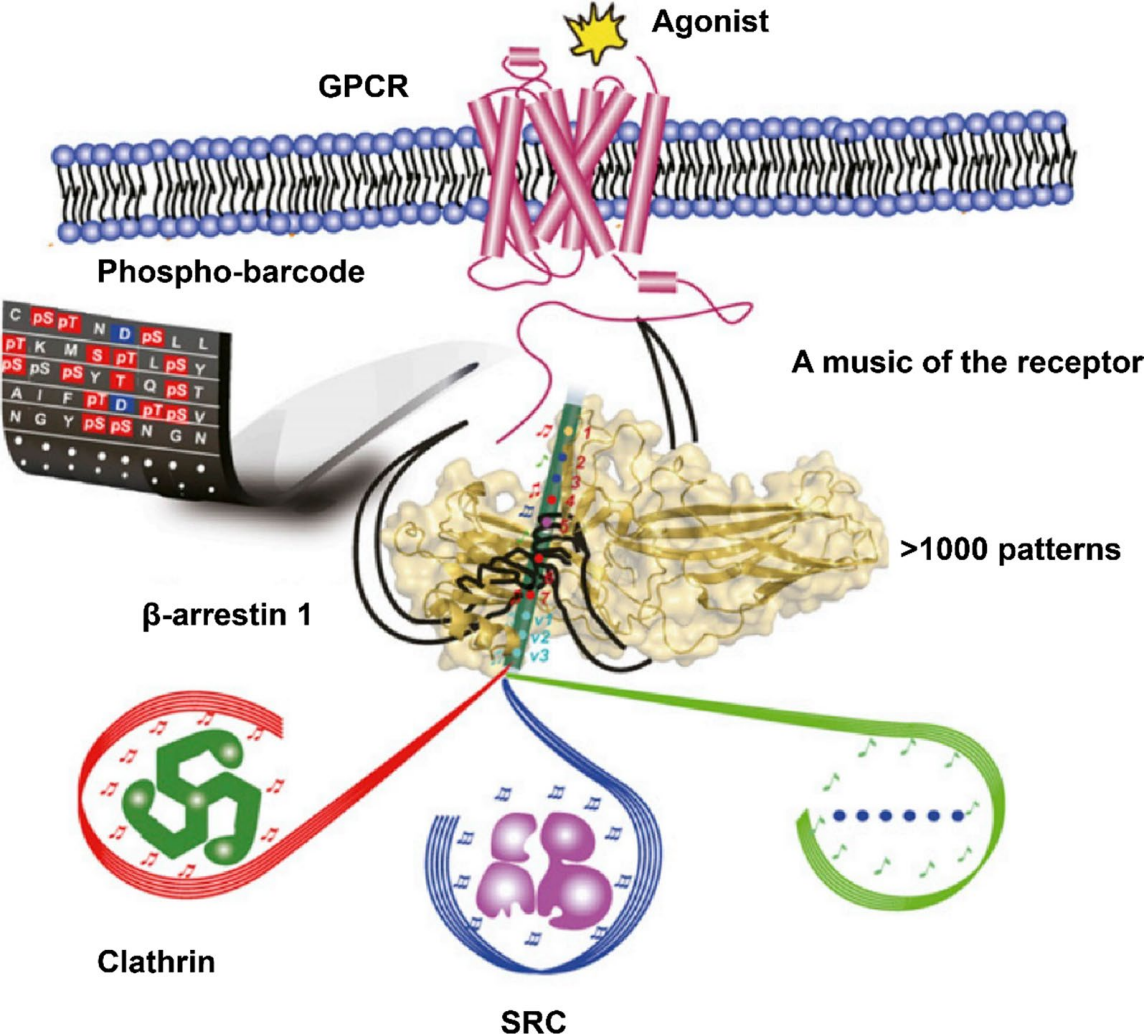
Flute model diagram. This picture is from the review by Yang et al. [28]

The bar code model explains the diversity of GPCR phosphorylation signaling. However, it is not clear how only a few arrestins and GRKs can transmit thousands of GPCR phosphorylation signals. In 2015, Fan Yang et al*.* proposed a flute model of GPCR phosphorylation signaling (Fig. 4) [27]. Using unnatural amino acid incorporation and fluorine-19 nuclear magnetic resonance (^19^F-NMR) spectroscopy, the researchers described the diverse phosphorylation signals of GPCRs from the perspective of molecular structure. Different GRKs catalyze phosphorylation at distinct sites, and β-arrestin recognizes phosphorylated sites in different structures, finally achieving specific signaling pathways. Seven phosphorylation sites (p1–p7) were detected in the V_2_R/β-arrestin1 crystal complex. GRK2 mainly catalyzes p1, p4, p6 and p7 phosphorylation. Β-Arrestin1 could identify these sites and recruit clathrin. GRK6 catalyzes p1 and p5 phosphorylation, enabling β-arrestin1 to attract SRC. The main role of clathrin is to mediate receptor endocytosis [112]. As a protein kinase, SRC plays an important role in cell morphology, proliferation, movement and survival [113].

Recently, Qing-Tao He et al. designed four V_2_R C-termini with different phosphorylation sites and analyzed the binding ability, structural changes and functional differences in relation to β-arrestin1 [31]. V_2_Rpp-1, V_2_Rpp-3, V_2_Rpp-4, V_2_Rpp-6, and 7 short peptides were designed according to the 7 phosphorylation sites (Thr347, Ser350, Ser357, Thr360, Ser362, Ser363, Ser364) at the C-terminus of V_2_R. The suffixed number represents the position of the C-terminal phosphorylation site. V_2_Rpp-1 not only activated β-arrestin but also recruited c-Raf-1 kinase to the V_2_Rpp-1-β-arrestin1 complex. The V_2_Rpp-3-β-arrestin1 complex interacted with MEK1. Both c-Raf-1 and MEK1 are effectors of V_2_R. V_2_Rpp-6,7 was mainly responsible for regulating the nuclear localization sequence (NLS) structure in the N-terminal domain of β-arrestin1. An analysis of the structure of V_2_Rpp-4 and β-arrestin1 showed that this complex causes the rearrangement of C-terminal pS357 and pT359 and causes β-arrestin1 and pT359 to form phospho interactions. In the study of V_2_R C-terminal phosphorylation sites, T359 was not included as one of the seven phosphorylation sites studied by the flute model [27, 28, 31]. However, this site was included in the bar code model studies [29, 30]. This is a primary difference between the two models in the study of V_2_R.

The flute model is similar to the bar code model; however, the flute model not only enriches the bar code model theory but also explains the GPCR phosphorylation recognition theory from the perspective of structural changes.

### GPCR phosphorylation QR code model

There are more than 800 known GPCRs in the human body, but research on receptor phosphorylation recognition patterns has mainly focused on rhodopsin and V_2_R [27, 29, 30, 97]. Ligands, GPCR type, GRK, arrestin, the phosphorylation sites may all affect phosphorylation recognition. These factors play a combined role to ultimately determine the biological impact of phosphorylation (Table 1). For instance, angiotensin II (Ang II) is the agonist for AT1R. Under the stimulation of Ang II, the activation of AT1R is maintains operational for only a short period of time. β-arrestin recruited to the AT1R phosphorylated by GRK2/3 and promoted AT1R endocytosis; however, ERK activation mediated by β-arrestin was enhanced when AT1R was phosphorylated by GRK5/6 [78]. AT1-AA was another agonist for AT1R. Under the stimulation of AT1-AA, AT1R was sustained activation, making the recruitment of β-arrestin restricted and the endocytosis of AT1R thus limited [35].

**Table 1:** Examples of multiple factors affecting GPCR phosphorylation recognition

Ligand	GPCR	GRK	Arrestin	Sites	Function	References
ISO	β_2_-AR	GRK2	β-arrestin2	T360, S364, S396, S401, S407, S411	Receptor desensitization and internalization; ERK activation	[79]
		GRK6		S355, S356		
Angiotensin II	AT1R	GRK2/ GRK3	β-arrestin	–	Receptor internalization	[78]
		GRK5/ GRK6			ERK activation	
Arginine-vasopressin	V_2_R	–	β-arrestin2	S357	Receptor trafficking	[30]
				S362, S363	Receptor trafficking; ERK activation	
				T360		
Dopamine	D2 dopamine receptor	GRK2/ GRK3	β-arrestin	S285, S286, T287, S288, T293, S311, S317, S321	Receptor trafficking and recycling	[128]
Carbachol	m2 Muscarinic acetylcholine receptors	GRK2	β-arrestin	Cluster S286-S290	Receptor internalization	[129–131]
				Cluster T307-S311	Receptor desensitization and internalization	
Carbachol	m3 Muscarinic acetylcholine receptors	GRK2	–	Cluster S331-S333, Cluster S348-S351	Receptor internalization	[132]
Methacholine		–	β-arrestin	S384		[133]

A similar phenomenon exists in the β_1_-AR. The β-adrenergic receptor agonist isoproterenol (ISO) activates receptors and signals via the Gs pathway [17]. Phosphorylated β_1_-AR recruits β-arrestin and drives receptor desensitization and endocytosis [114]. Meanwhile, the presence of β_1_-adrenergic receptor autoantibody (β_1_-AA) in patients with cardiovascular disease, although also an agonist of β_1_-AR, allows for sustained activation of β_1_-AR. Although it increases β_1_-AR phosphorylation, it inhibits the recruitment of the receptor to β-arrestin [115]. The reason for this needs further investigation. We speculate that it may be related to a mismatch of phosphorylation sites. That is, the β_1_-AR phosphorylation site is not efficiently recognized by β-arrestin under the action of β_1_-AA. Carvedilol as a representative of β-blocker could biased active β_1_-AR-Gi-β-arrestin pathway. Activation of this pathway could then exert cardioprotective effects by transactivating the epidermal growth factor receptor (EGFR)- extracellular signal-regulated kinase (ERK) pathway [49, 116]. The above evidence illustrates the phosphorylation of GPCR and its complex and variable functions. Therefore, only the precise regulation of the multiple factors involved in this process can ensure the accuracy of signaling.

**Fig. 5:**
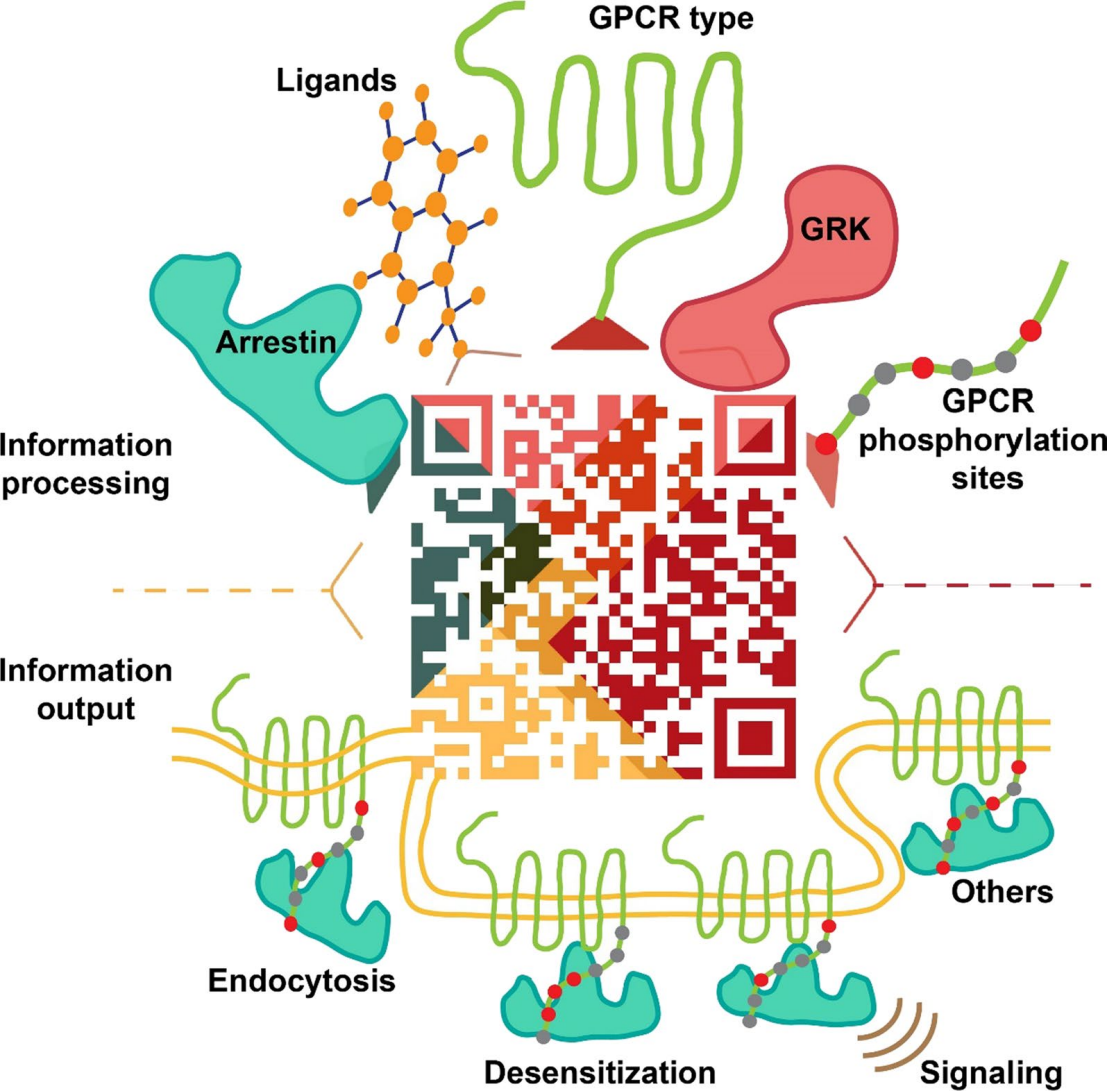
QR code model for GPCR phosphorylation recognition. Schematic diagram of the QR code model: ligands, GPCR type, GRK, arrestin, GPCR phosphorylation sites and other factors work together to determine the fate of phosphorylated receptor desensitization, endocytosis, the continuation of signal transmission, or other functions

This modulation is similar to a “QR” code (Fig. 5), which produces the final information after integrating various components. Ligands, the GPCR type, the GRK, arrestin and GPCR phosphorylation sites can all be thought of as information that is integrated into a QR code. Receptor desensitization, endocytosis, signaling and other functions represent information that is generated by the QR code.

## Conclusion

Accurately understanding the process of GPCR phosphorylation recognition will help to fully describe the way in which arrestin binds to receptors and its functional mechanism. It will also help us to better understand the physiological state of the body and provide potential targets and ideas for disease prevention and treatment. At present, research on the mode of GPCR phosphorylation recognition mainly focuses on the bar code model and the flute model [28–30, 62, 79]. These two models show the process of receptor phosphorylation recognition from the perspective of GPCRs, GRK, and arrestin, laying the foundation for GPCR phosphorylation recognition.

Analysis of the barcode model and the flute model revealed that the detection of the phosphorylation recognition process by these two models was mainly between GPCR and GRK or GPCR and arrestin [28, 29], with less detection of the mutual recognition between the three [79]. Our knowledge of the two recognition models is mainly focused on V_2_R and rhodopsin, with V_2_R being the most studied receptor carried out [27, 29, 30, 97]. But this is less than one fourth of one percent compared to the more than 800 GPCRs in the human body. This indicates that our recognition of GPCR phosphorylation patterns is still lacking. During receptor phosphorylation, factors such as ligand, receptor, GRK, arrestin, and phosphorylation site all affect the final function of the receptor. Therefore, we are proposing the QR code model to theoretically explain the effect of multiple factors acting together on receptor function.

The difference between the QR code model and the barcode model or the flute model is that the QR code model integrates the factors influencing the whole process from GPCR activation to arrestin function, such as ligands, GRK, etc. This model is developed on the existing theory and is based on the two existing recognition models. Based on our re-thinking on the recognition model of GPCR phosphorylation, we find both of the earlier models hold their own strengths, but limited. The barcode model and the flute model can determine the interactions between GPCR and GRK or arrestin with experimental precision, while the QR code model considers the receptor phosphorylation process macroscopically and is more theoretically oriented.

We are also conscious of the fact that the QR code model still has limitations and does not fully explain some specific phenomena of the GPCR phosphorylation recognition process. (1) Phosphorylation of some receptors is not involved in receptor endocytosis. Human lutropin receptor belongs to the class A GPCRs [117]. Although its agonist, human chorionic gonadotropin, can cause β-arrestin2-mediated receptor endocytosis, it is the receptor activity rather than the phosphorylation of the receptor that determines receptor endocytosis [118]. It is showed that mutations in the extracellular second loop of the receptor, F515A and T521A, enhance agonist-induced receptor endocytosis, while mutations in S512A and V519A impair agonist-induced receptor endocytosis. However, agonists have no effect on phosphorylation of either mutant receptor [119]. A comparative study of mutant receptors with impaired activity and impaired phosphorylation revealed that it is the activity of the receptor, but not the impaired phosphorylation, that that lengthens the time of receptor endocytosis [120]. (2) The presence of basal activation state of some receptors. The basal activation state of β_2_-AR in cardiomyocytes maintains cardiomyocyte beating without the involvement of ligands [17, 121]. (3) Intracellular activation of GPCR. β_1_-AR is distributed in both cell and organelle membranes and both can signal through the cAMP-PKA pathway [122, 123]. However, how the activated GPCR on the organelle membrane is desensitized and endocytosed remains to be further investigated. The above phenomena again illustrate the complexity of the GPCR signaling process. The existing theories or hypotheses are only summaries of known experimental results and speculations on the unknown. Our knowledge and understanding of GPCR phosphorylation recognition patterns still need to be further improved.

GPCR signal recognition, including phosphorylation, is the basis for the development of biased drugs. The presentation of allosteric microprocessors theory elaborated the mechanism of GPCR biased signaling. In this theory, biased ligands, GPCRs, transducers such as GRKs, G proteins and arrestins interacted allosterically. Biased ligands transmitted distinct GPCRs structural information that was processed into distinct biological outputs [124]. A biased agonist is a ligand that preferentially activates one receptor signaling pathway over another [37]. Oliceridine, a biased agonist of opioid receptors, specifically activates the μ-opioid receptor-G protein pathway and inhibits the binding of β-arrestin to the receptor, thereby achieving an analgesic effect. Unlike morphine, another agonist of μ-opioid receptors, the biased activation of oliceridine achieves an analgesic effect while avoiding adverse reactions such as respiratory depression caused by β-arrestin activation [125, 126]. Recent studies have also found that carvedilol, which has biased characteristics to activate the β_2_-AR-β-arrestin pathway, enhanced skeletal muscle contractility in mice without causing skeletal muscle cell hypertrophy. This feature might have therapeutic value for patients with sarcopenia and frailty. Although clenbuterol, another agonist of β_2_-AR, also enhanced the contractility of skeletal muscle cells, it caused cell hypertrophy and caused side effects such as arrhythmia [127]. Biased activation of the desired signaling pathway can minimize side effects while achieving the therapeutic effect, which is a characteristic of biased drugs. GPCR signaling pathways have been intensely studied for drug research and development owing to their characteristics of phosphorylation, and select pathways can be accurately activated or blocked to treat diseases.

## Data Availability

Not applicable.

## Abbreviations

GPCRs: G protein-coupled receptors
GRK: G protein-coupled receptor kinase
G proteins: Guanine nucleotide-binding proteins
AC: Adenylate cyclase
cAMP: Cyclic adenosine monophosphate
PKA: Protein kinase A
β_1_-AR: β_1_-Adrenoceptor
β_2_-AR: β_2_-Adrenoceptor
V2R: Vasopressin type 2 receptor
AT1R: Angiotensin II type I receptor
AT1-AA: Autoantibodies
α7 nAChR: α7 Nicotinic receptor
SD: Sprague Dawley rats
Δ9-THC: Δ9-Tetrahydrocannabinol
CB1: Cannabinoid receptor 1
ERK: Extracellular regulated protein kinases
PTH: Parathyroid hormone
NAMs: Negative allosteric modulators
PAMs: Positive allosteric modulators
SAMs: Silent allosteric modulators
AIDS: Acquired Immune Deficiency Syndrome
HIV-1: Human immunodeficiency virus type 1
siRNA: Small interfering RNA
MAPK: Mitogen-activated protein kinase
NTS1: Neurotensin receptor subtype 1
cDNA: Complementary deoxyribo nucleic acid
19F-NMR: Fluorine-19 nuclear magnetic resonance
NLS: Nuclear localization sequence
β_1_-AA: β_1_-Adrenergic receptor autoantibody
EGFR: Epidermal growth factor receptor
Ang II: Angiotensin II
